# Educational attainment trajectories among children and adolescents with depression, and the role of sociodemographic characteristics: longitudinal data-linkage study

**DOI:** 10.1192/bjp.2020.160

**Published:** 2021-03

**Authors:** Alice Wickersham, Hannah Dickson, Rebecca Jones, Megan Pritchard, Robert Stewart, Tamsin Ford, Johnny Downs

**Affiliations:** 1 Department of Psychological Medicine, Institute of Psychiatry, Psychology & Neuroscience, King's College London, UK; 2 Department of Forensic and Neurodevelopmental Science, Institute of Psychiatry, Psychology & Neuroscience, King's College London, UK; 3 Division of Psychiatry, University College London, UK; 4 Department of Psychological Medicine, Institute of Psychiatry, Psychology & Neuroscience, King's College London; and South London and Maudsley NHS Foundation Trust, London, UK; 5 Department of Psychiatry, University of Cambridge, Cambridge, UK; 6 Department of Child and Adolescent Psychiatry, Institute of Psychiatry, Psychology & Neuroscience, King's College London; and South London and Maudsley NHS Foundation Trust, London, UK

**Keywords:** Depressive disorders, education and training, cohort study, trajectories, childhood and adolescence

## Abstract

**Background:**

Depression is associated with lower educational attainment, but there has been little investigation of long-term educational trajectories in large cohorts with diagnosed depression.

**Aims:**

To describe the educational attainment trajectories of children with a depression diagnosis in secondary care, and to investigate whether these trajectories vary by sociodemographic characteristics.

**Method:**

We identified new referrals to South London and Maudsley's NHS Foundation Trust between 2007 and 2013 who received a depression diagnosis at under 18 years old. Linking their health records to the National Pupil Database, we standardised their performance on three assessments (typically undertaken at ages 6–7 years (school Year 2), 10–11 (Year 6) and 15–16 (Year 11)) relative to the local reference population in each academic year. We used mixed models for repeated measures to estimate attainment trajectories.

**Results:**

In our sample of 1492 children, the median age at depression diagnosis was 15 years (interquartile range = 14–16). Their attainment showed a decline between school Years 6 and 11. Attainment was consistently lower among males and those eligible for free school meals. Black ethnic groups also showed lower attainment than White ethnic groups between Years 2 and 6, but showed a less pronounced drop in attainment at Year 11.

**Conclusions:**

Those who receive a depression diagnosis during their school career show a drop in attainment in Year 11. Although this pattern was seen among multiple sociodemographic groups, gender, ethnicity and socioeconomic status predict more vulnerable subgroups within this clinical population who might benefit from additional educational support or more intensive treatment.

Depression is a common mental disorder among children and adolescents,^1^ and in this age group is characterised by symptoms such as low mood, irritability, reduced energy and cognitive problems.^2,3^ Previous longitudinal work has shown an association between depression and subsequent lower educational attainment; for instance, children and adolescents with more symptoms of depression receive lower grades, are less likely to complete secondary education and are less likely to enrol in college.^4–6^ However, attainment is unlikely to remain stable across development, and can change throughout a pupil's school career, yet little is known about depression and changes in attainment over time. Additionally, these attainment patterns may not be the same for all children with depression. Sociodemographic characteristics such as gender, ethnicity and socioeconomic status have been found to predict both attainment and the presence of emotional disorders in the general population.^1,7–10^ These factors are therefore important to investigate in predicting the attainment patterns of children with depression. Understanding the educational attainment trajectories of children with depression may highlight the need for both mental health and educational interventions at key developmental stages and highlight particularly disadvantaged sociodemographic groups as priority.

Previous longitudinal work in the field of depression and attainment has tended to use small, community-based samples, and often relied on self- or informant-reported measures of depression and attainment. A recent study by Rahman *et al* successfully leveraged linked health and education data from Wales to investigate educational attainment as a predictor of depression onset.^11^ Their findings were suggestive that children and adolescents may show declines in school performance before receiving a diagnosis of depression, although their use of dichotomised attainment data precluded a more granular investigation of educational attainment trajectories, and the role of sociodemographic characteristics in attainment changes was not fully explored.^12^ To investigate this further, we used mixed models to investigate changes in school performance among children with depression using linked administrative health and education records for a large clinical cohort. The primary aims were to describe the educational attainment trajectories of children who received a diagnosis of depression and to investigate whether these trajectories varied according to their sociodemographic characteristics. As this work was exploratory, no specific hypotheses were proposed.

## Method

This study followed RECORD reporting guidelines (supplementary Table 1, available at https://doi.org/10.1192/bjp.2020.160).^13^

### Design and sample

We carried out a historical, longitudinal cohort study that made use of a linked health and education administrative data-set. The South London and Maudsley NHS Foundation Trust Biomedical Research Centre (SLaM BRC) Case Register contains pseudonymised electronic health records (EHRs) from secondary mental health services, including child and adolescent mental health services (CAMHS).^14^ These CAMHS records have been linked to the National Pupil Database (NPD), which contains results on national assessments for all pupils in England's state schools.^15^ Linkage was undertaken at the individual level using fuzzy and deterministic data linkage approaches based on personal identifiers (names, dates of birth and postcodes).^16,17^ The Clinical Record Interactive Search (CRIS) data resource, including the linked data here, has received research ethics approval for secondary analyses (Oxford Research Ethics Committee C, reference 18/SC/0372).

We ascertained all new referrals to SLaM who received a first diagnosis of depression within the observation window of 1 January 2007 to 31 December 2013. A depression diagnosis was defined as any ICD-10 F32x depressive disorder in the EHR structured primary or secondary diagnosis fields.^18^ Cohort members were required to be below 18 years old at diagnosis, and their closest registered address to diagnosis date to be within the SLaM catchment area (London boroughs of Croydon, Lambeth, Lewisham and Southwark).

### Outcome

Educational attainment was measured from the NPD. In England, statutory testing typically takes place at three key time points: Year 2 (key stage 1, typically assessed at age 6–7), Year 6 (key stage 2, age 10–11) and Year 11 (key stage 4, age 15–16). In accordance with scoring produced by the Department for Education, we used average point scores for Year 2 (based on teacher-assessed reading, writing, maths and science) and Year 6 (based on English and maths national tests).^19,20^ For Year 11 we used capped total point scores based on each pupil's best eight General Certificate of Secondary Education (GCSE) or equivalent grades.^21^ In line with previous research, at each time point we converted these point scores into standardised scores.^22^ Attainment scores for our sample were standardised using the mean and standard deviation of attainment scores from all available NPD records for a local reference population. This local reference population included children and adolescents who were resident in the SLaM catchment area between 2007 and 2013, and those who were referred to SLaM CAMHS from either inside or outside the SLaM catchment area (*n* = 276 655). Standardisation using those in the local reference population who had available attainment data was carried out separately for each academic year, to account for secular changes in test scoring over time.

### Exposures

Gender and ethnicity were recorded in EHRs. In cases where gender was missing from EHRs, it was assigned probabilistically from the NPD at each of the three school years, taking the gender most frequently recorded. In cases where ethnicity was missing from EHRs, it was assigned from NPD school census records. Finally, in cases where ethnicity remained missing, SLaM patient notes were manually searched for key words indicating ethnicity. For this study, ethnicity was categorised as White, Black, Asian, mixed or Other. ‘Other’ ethnicity consisted of Chinese and other unspecified ethnic groups (supplementary Table 2).

In the UK, eligibility for free school meals (FSM) is determined on the basis of low parental income and is therefore widely used as a proxy for socioeconomic disadvantage.^23^ It is recorded in several school census and absence variables in the NPD. To minimise missing data, we combined all available FSM variables to indicate whether each pupil had ever been recorded as eligible for FSM.

### Covariates

Age at diagnosis was included as a covariate and calculated from EHRs using each child's birth date and the date of first depression diagnosis. Neurodevelopmental disorder was also explored as a covariate in a supplementary analysis and was defined as any record of an intellectual disability (F70–F79), pervasive developmental disorder (F84) or hyperkinetic disorder (F90) during the observation window.

### Data analysis

Logistic regression analyses were conducted to investigate whether the sociodemographic characteristics of the cohort predicted missingness in attainment data. A linear mixed model for repeated measures was fitted to examine changes in educational attainment between Years 2, 6 and 11, and further investigated using post-estimation commands. Linear mixed models are a generalisation of linear regression able to incorporate both fixed and random effects, thereby permitting modelling of repeated measures data without violating assumptions of independence.^24^ For example, correlations between educational attainment measures for the same pupil at different time points can be accounted for using a random effect of pupil. Attainment trajectories were modelled with the main predictors as assessment year (specified as an indicator variable), gender, ethnicity, FSM eligibility, and interaction terms between each sociodemographic characteristic and assessment year. All sociodemographic characteristics were included in the same model to control for each other's effects, as there are known interrelationships between gender, ethnicity and deprivation in predicting educational attainment.^9,10^ The model controlled additionally for age at depression diagnosis. The model was fitted using maximum likelihood estimation (MLE) and we specified an unstructured residual-error covariance matrix, to allow the residual variances for different school years and the covariances between pairs of school years to be estimated separately. Finally, *post hoc* supplementary analyses were conducted to investigate the role of comorbid neurodevelopmental disorder and age at diagnosis. We re-ran the longitudinal model making an additional adjustment for neurodevelopmental disorder. We also re-ran the longitudinal model three times, each time fitting an additional three-way interaction term between age at diagnosis, assessment year and one of the socio-demographic characteristics. Analyses were conducted in Stata 15.1 for Windows.^25^

## Results

### Missing data

In total, 1767 children were ascertained who were eligible for inclusion from their mental health EHR data, 1514 (85.7%) of whom had been successfully linked to NPD records. Of these, 1492 (98.5%) had available attainment data for at least one time point (supplementary Fig. 1). We conducted a logistic regression analysis to assess predictors of successful linkage and attainment data availability (supplementary Table 3). Compared with eligible children who were not linked to the NPD or did not have any available attainment data, those in the study sample were younger at diagnosis, less likely be male and less likely to be from Asian or Other ethnic backgrounds. These variables similarly predicted missingness of attainment scores at each assessment time point among eligible children, although the effect of age at diagnosis reversed direction over time; those who received their diagnosis at an older age were less likely to have available Year 2 and 6 attainment data, but were more likely to have available Year 11 attainment data (supplementary Table 4).

In the final study sample (*n* = 1492), standardised and continuous attainment scores were missing for *n* = 190 (12.7%) in Year 2, *n* = 141 (9.5%) in Year 6 and *n* = 391 (26.2%) in Year 11. For the remaining variables of interest, there were either no missing data (gender, age at diagnosis, neurodevelopmental disorder) or missing data rates were very low (1.7% for FSM eligibility and 0.7% for ethnicity; 2.4% in total). Children with missing attainment data at one or two of the three time points were included in modelling, but the small number who were missing data on ethnicity or FSM eligibility were excluded from the linear mixed model.

### Descriptive statistics

**Fig. 1:**
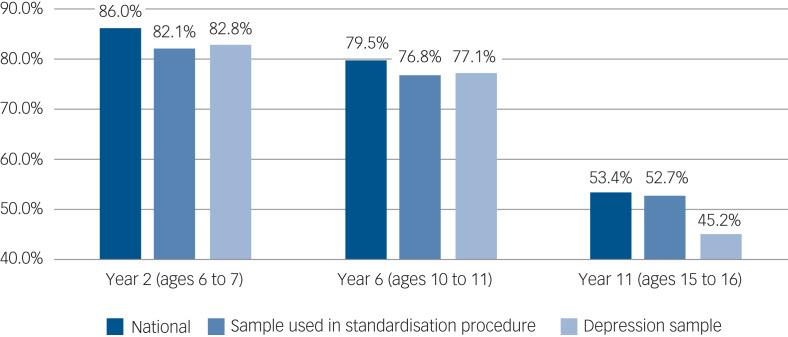
Proportion of study sample meeting expected attainment thresholds at each time point, compared with all available National Pupil Database records (as used in standardisation procedures for the present study), and national figures from 2010 (the midpoint of our observation window).

The majority of the study sample was female (*n* = 1068, 71.6%). Slightly under half of the children had at some time been eligible for FSM (*n* = 652, 44.5%). Most were from a White ethnic background (*n* = 818, 55.2%), followed by Black (*n* = 353, 23.8%), mixed (*n* = 159, 10.7%), Other (*n* = 79, 5.3%) and Asian (*n* = 73, 4.9%). The median age at depression diagnosis was 15 years (interquartile range = 14–16). Most of the study sample met the expected attainment threshold of level 2 or above in Year 2 writing (*n* = 1014, 77.7%), reading (*n* = 1058, 81.1%), maths (*n* = 1112, 85.5%) and science assessments (*n* = 1128, 86.7%). Most also met the expected threshold of level 4 or above in Year 6 English (*n* = 1092, 81.3%) and maths assessments (*n* = 991, 72.8%). Attainment in Years 2 and 6 appeared to be lower than national levels but was slightly higher than levels seen in the local reference population used for standardisation procedures (Fig. 1). However, only *n* = 501 (45.2%) met the expected threshold of five A*–C GCSE or equivalent grades (including English and maths) in Year 11, much lower than the proportion meeting this threshold in both national estimates (53.4%) and in the local reference population (52.7%).

National data were sourced from Department for Education 2010 statistical releases.^19,20,26^ Year 2 percentages are a mean average across maths, reading, writing and science; Year 6 percentages are a mean average across English and maths.

### Educational attainment trajectories

For the reference group (female, White, ineligible for FSM and age 15 at depression diagnosis), attainment was stable between Years 2 and 6 (estimated *z*-score change = 0.03, 95% CI −0.04 to 0.09, *P* = 0.425), but there was strong evidence for a decline in attainment between Years 6 and 11, as standardised relative to the rest of the local population in each academic year (estimated *z*-score change = −0.52, 95% CI −0.63 to −0.41, *P* < 0.001).

**Fig. 2:**
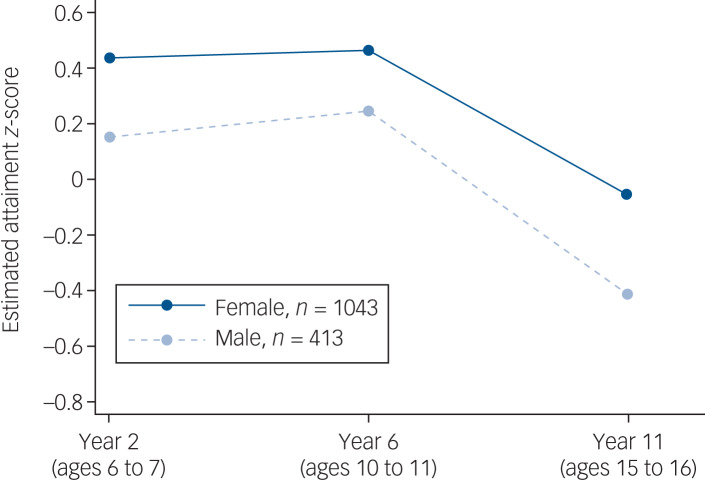
Educational attainment trajectories by gender, as estimated from the longitudinal mixed model.

**Fig. 3:**
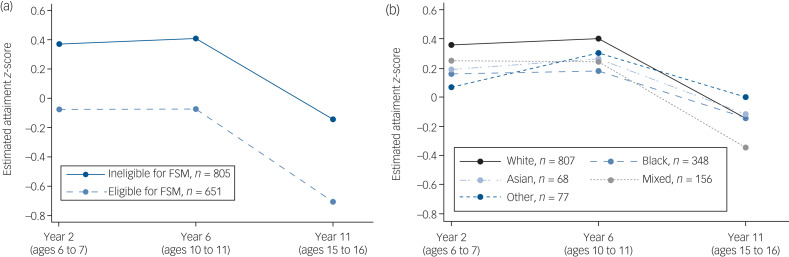
Educational attainment trajectories by (a) eligibility for free school meals (FSM) and (b) ethnicity, as estimated from the longitudinal mixed model.

There was strong evidence for lower attainment among males than females at all three time points for those with depression (Table 1 and supplementary Table 5). However, the pattern of change in attainment over time was similar for both genders, with no evidence of an interaction between gender and assessment year (Fig. 2). Similarly, there was strong evidence for consistently lower attainment among those eligible for FSM compared with those who were not at all three time points, with similar changes in attainment over time in both groups and likewise no evidence of an interaction between FSM eligibility and assessment year (Fig. 3).

**Table 1:** Estimated attainment *z*-scores by sociodemographic group at each time point^a^ and differences in rate of change over time between sociodemographic groups

	Year 2 estimate (ages 6–7), *z*-score difference (95% CI)	Year 6 estimate (ages 10–11), *z*-score difference (95% CI)	Year 11 estimate (ages 15–16), *z*-score difference (95% CI)	Evidence for a difference in the rate of change between Year 2 and Year 6, interaction *P*	Evidence for a difference in the rate of change between Year 6 and Year 11, interaction *P*
Gender					
Female	Reference group	–	–	–	–
Male	−0.29 (−0.39 to −0.18)	−0.22 (−0.33 to −0.11)	−0.36 (−0.51 to −0.20)	0.152	0.081
Free school meal eligibility					
Ineligible	Reference group	–	–	–	–
Eligible	−0.44 (−0.54 to −0.34)	−0.48 (−0.58 to −0.38)	−0.56 (−0.70 to −0.42)	0.330	0.255
Ethnicity					
White	Reference group	–	–	–	–
Black	−0.20 (−0.32 to −0.08)	−0.22 (−0.34 to −0.10)	0.01 (−0.16 to 0.18)	0.648	0.008
Asian	−0.17 (−0.42 to 0.08)	−0.14 (−0.38 to 0.11)	0.04 (−0.30 to 0.37)	0.733	0.316
Mixed	−0.11 (−0.27 to 0.05)	−0.16 (−0.32 to −0.00)	−0.20 (−0.42 to 0.03)	0.453	0.743
Other	−0.29 (−0.53 to −0.06)	−0.10 (−0.32 to 0.12)	0.15 (−0.16 to 0.46)	0.039	0.116

a. Each estimate is shown at the level of the reference category for the other sociodemographic covariates and at the median age at diagnosis of depression (age 15 years).

The trajectories are shown at the level of the reference category for ethnicity (White) and eligibility for free school meals (ineligible), and at the median age at diagnosis of depression (age 15 years).

In each graph, the trajectories are shown at the level of the reference category for the other sociodemographic covariates, and at the median age at diagnosis of depression (age 15 years).

No differences were observed in the educational trajectories of Asian and mixed ethnic groups relative to White ethnic groups. However, compared with White ethnic groups, we found lower attainment among Black and Other ethnic groups in Year 2, and among Black ethnic groups in Year 6. Interactions between ethnicity and assessment year were present, such that the observed changes in attainment over time were not the same for all ethnic groups (Fig. 3). Compared with individuals from White ethnic groups, those from Black ethnic groups had a less pronounced decline in their attainment after Year 6, such that there was no longer evidence that they had lower attainment than White ethnic groups in Year 11. There was also evidence that Other ethnic groups showed an improvement between Years 2 and 6 compared with White ethnic groups.

### Supplementary analyses

One possible explanation for the different trajectories is variation in the rates of neurodevelopmental disorders among the different sociodemographic groups. A small proportion had record of a neurodevelopmental disorder during the observation window (*n* = 86, 5.8%), and this proportion varied slightly according to sociodemographic group, most notably gender (males *n* = 44, 10.4%; females *n* = 42, 3.9%). Nonetheless, no material changes were observed in results from linear mixed modelling after additionally adjusting for neurodevelopmental disorder (full results available from corresponding author on request).

Another possible explanation for the different trajectories is that the sociodemographic characteristics under study are associated with different ages at depression diagnosis. Therefore, we conducted *post hoc* supplementary analyses to further investigate whether age at diagnosis interacted with gender, ethnicity and FSM eligibility to predict attainment trajectories. We re-ran the longitudinal model three times, each time fitting an additional three-way interaction term between age at diagnosis, assessment year and one of the sociodemographic characteristics. Findings showed no evidence to support these three-way interactions, and likelihood-ratio tests did not indicate that the addition of these interaction terms improved model fit (full results available from corresponding author on request). Therefore, age at diagnosis did not appear to modify the effects of gender, ethnicity or FSM eligibility on the observed attainment trajectories.

## Discussion

In this study, we investigated the educational attainment trajectories of a clinical cohort of children and adolescents who received a depression diagnosis. We found that, relative to a local reference group, performance on tests was stable between Year 2 (typically assessed at ages 6–7) and Year 6 (ages 10–11), but significantly declined in Year 11 (ages 15–16). The proportion of our sample meeting expected attainment thresholds in Years 2 and 6 was comparable with, and sometimes exceeded, national and local estimates, but only 45.2% of our sample with available Year 11 data received five A*–C GCSE or equivalent grades, compared with 53.4% nationally in 2010.^26^ Moreover, this pattern of stability followed by decline was found across many different sociodemographic groups, the only exception being that some ethnic groups followed slightly different attainment trajectories. For example, Black ethnic groups showed lower attainment than White ethnic groups between school Years 2 and 6 but showed a less pronounced drop in attainment at Year 11.

### Strengths and limitations

The observed changes in school performance over time highlight the importance of measuring educational outcomes at multiple time points, rather than focusing on single estimates, as some longitudinal work has previously done. The attainment measures used in this study overcome the reporting biases associated with self- and informant-rated measures. However, because assessments were not identical at each time point, it was necessary to standardise attainment at each time point relative to a local reference population in each academic year group. This adds complexity to the interpretation of our results, which has led some methodologists to discourage the use of standardised scores in longitudinal studies.^27^ However, in the UK educational system, and indeed in many countries, attainment is itself measured in terms of how children perform relative to expected national standards, rather than relative to each child's individual starting points.^28^ In light of this emphasis in educational policy, we felt that it would be more illuminating to analyse attainment as a relative construct.

We had access to individual-level data only for a group of children and adolescents who were either local to SLaM, or had been referred to SLaM services from outside the catchment area. Therefore, this was the reference group used for standardisation procedures. An advantage of this is that attainment among the depression cohort was scored relative to a reasonably comparable group: compared with the rest of England, the SLaM catchment area has a higher proportion of minority ethnic groups and a higher proportion of both the highest and lowest socioeconomic groups.^14^ However, focusing on a more comparable reference population derived from SLaM and its catchment area limits generalisability to other areas and the UK more generally.

This study overcomes some drawbacks of traditional longitudinal studies, such as non-response and attrition biases. However, the use of linked administrative data presents some unique challenges. The sample can be limited by the scope of the data-sets, which further limit generalisability: for instance, our methods for identifying children with depression relied on clinicians consistently and accurately recording ICD-10 depression codes in EHR structured fields, and the NPD only captures children in state education.^15^ Diagnostic practices can vary between clinicians, and we were unable to conduct diagnostic validation work, or a more detailed analysis of the presence of depression symptoms in this cohort. Consequently, an understanding of whether presenting symptoms are associated with attainment trajectories should be an area for future research, particularly since symptom profiles may vary with age. It should also be noted that only children referred to CAMHS were captured by this work, limiting generalisability to the large number of children and adolescents who do not come into contact with mental health services for their disorder.^1^

Although the proportion of missing data was generally low in this study, the reasons for missed data links or incomplete records in administrative data are not always clear, making it difficult to investigate bias. Similarly, we calculated point scores in Years 2 and 6 in accordance with Department for Education tables to ensure that results were comparable with national publications from the time, but this requires that some educational outcomes be coded as missing when they may in fact imply low attainment; examples include disapplication from the national curriculum or being absent for assessments. However, only a small proportion of the study sample (1.7%) were absent or disapplied for any assessment in either assessment year, so this is unlikely to have materially biased analyses. We were also unable to investigate the possible effects of early or late exam taking; however, at each time point, >99% of the sample had ages consistent with being in school Years 2, 6 and 11 at the time of these assessments, as is typical in the UK, such that this again is unlikely to have materially biased analyses.

Our consideration of covariates was somewhat limited. For instance, our binary indicator for FSM status represents a moderately reliable but unnuanced proxy measure of socioeconomic status.^29^ Owing to small cell sizes, we were also unable to conduct more granular analyses relating to ethnicity, and the method of ascertaining ethnicity for administrative records can be varied and imprecise (for instance, ethnicity in EHRs might be self-reported or ascertained by a clinician). Finally, we were unable to consider other factors that might affect attainment trajectories, such as social or school factors and depression treatment.

### Comparison with previous literature

Our finding that a cohort of children who received a depression diagnosis at some point before age 18 showed an overall drop in their educational attainment is consistent with previous work showing poorer school outcomes among children with depressive symptoms.^4,6^ Although Rahman et al observed that depression was associated with a decline in performance throughout primary school, we found evidence of a drop in performance only after Year 6. This difference may be attributable to our use of mixed models and more granular educational attainment measures.^11^ Indeed, the average age at depression diagnosis in this cohort (15 years) coincided with the typical Year 11 assessment age (15–16 years), the age at which we observed a decline in attainment. While we cannot draw any causal inferences from this, previous longitudinal work has found that the course of attainment appears to mirror the course of concurrent depressive symptoms.^30,31^ In this context, our findings indicate that the onset of depression and difficulties resulting in lower attainment may coincide.

These findings also add to previous work on the potentially modifying effects of sociodemographic characteristics on the association between depression and educational attainment. Consistent with previous work in the general population, our findings showed lower overall attainment among boys, those from more deprived socioeconomic backgrounds and some ethnic groups.^7–10^ This did not appear to be attributable to varying ages at depression diagnosis or rates of neurodevelopmental disorders in different sociodemographic groups. Moreover, the pattern of stability in attainment followed by a decline in Year 11 was notably consistent for all of the sociodemographic groups under study, with the exception of the Black and Other ethnic groups. This suggests that the observed attainment drop in Year 11 among those with depression is robust and pervasive across children and adolescents with diverse characteristics. Future studies could investigate this further using attainment trajectories broken down by academic subject area. Conducting a more fine-grained analysis of ethnicity would also aid interpretation of the different trajectories we observed among Black and Other ethnic groups, as at present the reasons behind this are unclear.

### Implications of findings

In England, GCSE attainment is a key determinant of whether pupils enter further education and it remains important for some higher education courses. National estimates suggest that, in general, fewer pupils reach expected attainment thresholds in Year 11 than in Years 2 and 6, perhaps owing to increased academic demands in this assessment period. However, our finding that this cohort of children with depression saw a comparatively substantial decline in attainment in Year 11 is concerning. This may contribute to a long-term vicious cycle in the relationship between educational expectations, worry about performance, poor mental health, and poor educational and occupational outcomes.^11,32–34^ This may also have implications at the school level. Secondary school performance and accountability are primarily informed by Progress 8, a measure of pupils’ progress between the end of primary school and the end of secondary school. With depression likely to have a negative impact on Progress 8 scores, schools with higher incidences of depression may be disproportionately affected by the drop in attainment observed in this group. Additional educational support for children struggling with their mental health, particularly in the lead up to GCSEs, might attenuate the observed attainment drop. It might, for example, be advisable for some to consider deferring or staggering their exams. Mental health practitioners should discuss schoolwork and future plans with young people and their families and liaise with schools to plan around educational challenges.

Our results also reinforce known complexities associated with gender, ethnic and deprivation inequalities in educational attainment. Speculation on how sociodemographic inequalities might be addressed at the societal or school level is ongoing.^8–10^ Targeted educational support for children struggling with depression might particularly benefit boys and those from deprived backgrounds, who were particularly vulnerable subgroups in this study, although as already noted the observed drop in attainment at GCSE was pervasive across many groups, such that all children with depression might benefit from such support. Additionally, although we cannot infer a causal relationship from this study, the identification and treatment of depression prior to this key educational milestone might also be critical to improving attainment, as implied by previous reviews.^4,6^ School-based interventions may present a means of reducing depressive symptoms in the hope of managing the impact that these symptoms might have on attainment.^35^ This is in keeping with growing recognition in policy and practice that good mental health provision is an important component of effective educational systems.^36,37^

## Data Availability

The data that support the findings of this study are not publicly available but can be accessed with permissions from both the Department for Education and South London and Maudsley NHS Foundation Trust. A.W. and J.D. had full access to the study data. At the time of publication, access is ongoing.

## Supplementary material

For supplementary material accompanying this paper visit http://dx.doi.org/10.1192/bjp.2020.160. click here to view supplementary material
